# Knockdown of CCNB1 alleviates high glucose-triggered trophoblast dysfunction during gestational diabetes via Wnt/β-catenin signaling pathway

**DOI:** 10.1515/med-2024-1119

**Published:** 2025-01-13

**Authors:** Biru Xiao, Wenmiao Zhang, Nini Ji, Qiuyue Chen

**Affiliations:** Department of Obstetrical, The First Affiliated Hospital of Wenzhou Medical University, Wenzhou, Zhejiang, 325000, China; Department of Obstetrical, The First Affiliated Hospital of Wenzhou Medical University, Nanbaixiang Street, Ouhai District, Wenzhou, Zhejiang, 325000, China

**Keywords:** gestational diabetes mellitus, Cyclin B1, inflammation, glucose uptake, Wnt/β-catenin

## Abstract

Gestational diabetes mellitus (GDM), defined as glucose intolerance occurring or first detected during pregnancy, affects approximately 8% of pregnancies worldwide. The dysfunction of trophoblasts in pregnancies complicated by GDM is associated with changes in trophoblast cell functions, resulting in compromised proliferation and regulation of the cell cycle. Cyclin B1 (CCNB1), a pivotal controller of the start of mitosis, is crucial in these mechanisms. Nevertheless, the precise function of CCNB1 in trophoblast dysfunction related to GDM has not been extensively investigated. The aim of this study was to investigate CCNB1’s role in high glucose (HG)-triggered trophoblast. Herein, we revealed that in HG-stimulated HTR8/SVneo cells, CCNB1 is highly expressed. Knockdown of CCNB1 significantly promotes the growth of HG-stimulated HTR8/SVneo cells and suppresses inflammation (*p* < 0.05). Additionally, reducing CCNB1 expression significantly improves glucose uptake and inhibits the Wnt/β-catenin pathway in HG-stimulated HTR8/SVneo cells (*p* < 0.05). In conclusion, our study demonstrated that the deletion of CCNB1 can alleviate trophoblast dysfunction induced by HG in GDM through the Wnt/β-catenin pathway. This suggests that CCNB1 may be a potential target for managing GDM. Although our results underscore the potential therapeutic benefits of reducing CCNB1 in mitigating trophoblast dysfunction, it is important to note that the study is limited by its reliance on a single cell line and the absence of *in vivo* validation.

## Graphical abstract

**Figure:**
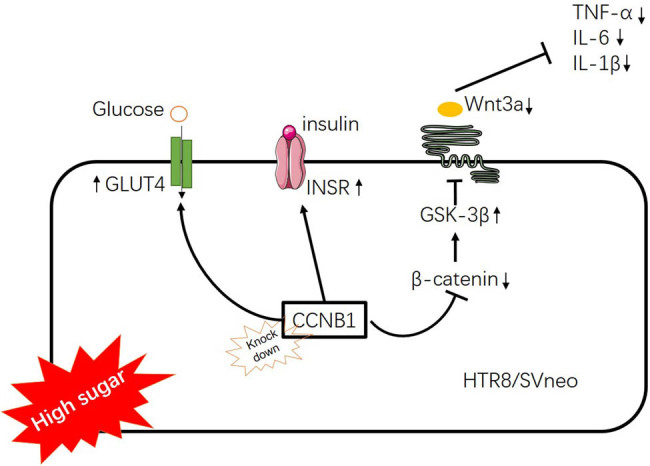

## 1 Introduction

During pregnancy, gestational diabetes mellitus (GDM) is characterized by the onset or first identification of glucose intolerance. It is considered a common complication of pregnancy [1]. The increasing prevalence of obesity and GDM during gestation can be attributed to modifications in lifestyle and eating habits [2]. Globally, around 8% of pregnancies are impacted by GDM, with variation in prevalence based on socioeconomic factors and diagnostic standards. The presence of GDM presents significant and potentially life-threatening dangers to both mothers and babies [3]. Research indicates that GDM significantly increases the rates of preeclampsia, macrosomia, and difficult deliveries while also contributing to spontaneous abortion, stillbirth, and congenital malformations [4]. Therefore, early diagnosis and timely therapeutic strategies are critical in reducing the risk of adverse pregnancy outcomes for GDM patients.

In GDM-complicated pregnancies, placentas exhibit trophoblast dysfunction. The hyperglycemic intrauterine environment in GDM affects both fetal and placental development [5]. Despite the lack of clarity on the precise mechanisms of GDM, research indicates that changes in trophoblast function, including differentiation, invasion, proliferation, apoptosis, and cell cycle control, may be significant factors [6]. Moreover, the Wnt/β-catenin signaling pathway is recognized as a critical modulator of trophoblast differentiation and placental growth. Increasing evidence suggests that dysregulation of this pathway is linked to the development of GDM [7].

Cyclin B1 (CCNB1) is a critical regulator of mitotic initiation, exhibiting cyclical expression throughout the cell cycle [8]. CCNB1 has been associated with controlling the cell cycle and DNA replication in hepatocellular carcinoma (HCC), suggesting possible targets for diagnosis and treatment [9,10]. Circ-CCNB1, a circular RNA originating from CCNB1, is involved in regulating trophoblast proliferation and invasion in cases of spontaneous abortion [11]. Furthermore, research has indicated a substantial increase in CCNB1 expression in diabetic mice in comparison to the control group [12]. Cyclin B1 is highly expressed in HCC, breast cancer, bladder cancer, and other cancers, promoting the proliferation and invasion of cancer cells [13,14,15]. Furthermore, there is a strong correlation between the atypical presentation of Cyclin B1 and the pathophysiology underlying cardiovascular and autoimmune disorders [16,17]. Nevertheless, the functions and pathways through which CCNB1 operates in GDM are yet to be adequately investigated.

The aim of this study is to investigate the role of CCNB1 in high glucose (HG)-induced trophoblast dysfunction during GDM. The primary aim is to investigate the potential of reducing trophoblast apoptosis, inflammatory response, and impaired glucose uptake induced by HG through modulation of the Wnt/β-catenin signaling pathway via CCNB1 knockdown. This study may lead to the development of targeted therapies aimed at improving pregnancy outcomes in GDM patients.

## 2 Materials and methods

### 2.1 Cell culture

Human HTR8/SVneo cells and BeWo cells were obtained from American type culture collection (USA) and maintained with Dulbecco’s modified eagle medium supplied with 10% fetal bovine serum at 37°C, 5% CO_2_. To establish a HG cell model, the cells were treated with elevated levels of glucose (30 mM d-glucose, Sigma, USA) for a period of 72 h.

### 2.2 Quantitative polymerase chain reaction (qPCR)

Tissue and cellular RNA were extracted with the Trizol reagent (TaKaRa, Japan). Subsequently, the total RNA was subjected to reverse transcription using the RT reagent Kit from Takara. Quantitative PCR analysis was carried out utilizing the SYBR Ex Taq™ II kit from Takara.

The used primers were listed as below: CCNB1: F: 5′-TGCAAAGGCAAGCAGTACAA-3′, R: 5′-GGTTGCTCCATGTACTGACC-3′, GAPDH: F: AGAAGGCTGGGGCTCATTTG, R: AGGGGCCATCCACAGTCTTC′.

### 2.3 CCK-8 assay

HTR8/SVneo cells were seeded into 96-well plates, and incubated. Then, cells were maintained and cultured with CCK-8 solution (C0038, Beyotime, China) for 4 h. Then, the OD450 value was measured.

### 2.4 EdU staining

Cells were treated with 50 μM EdU (Abcam, UK) for 2 h, fixed with 4% paraformaldehyde (PFA) and treated with glycine for 5 min. Cells were then stained with DAPI. The images were captured.

### 2.5 Cell apoptosis

Annexin V/PI apoptosis detection was carried out according to the instructions provided by the manufacturer for the identification of apoptosis (Sigma Aldrich, USA).

### 2.6 Immunostaining assay

Cells were fixed with 4% PFA, and blocked with 5% bovine serum albumin (BSA) in phosphate buffer TWEEN 20 (PBST), and then incubated with primary antibody targeting GLUT4 (1:300, ab33780, Abcam). Following the PBST wash, the cells were incubated with secondary antibodies labeled with Alexa 488 (Invitrogen, CA), while 4′,6-diamidino-2-phenylindole was used to stain the cell nuclei. Subsequently, images were captured using a fluorescent microscope.

### 2.7 ELISA

The level of TNF-α, IL-6, as well as IL-1β (Abcam, UK) in the culturing supernatant was assessed by ELISA kit in agreement with the suggestions of the manufacturer.

### 2.8 Immunoblotting

HTR8/SVneo cells were lysed for protein extraction. The extracted proteins were separated by sodium dodecyl sulfate polyacrylamide gel electrophoresis, and transferred to poly(vinylidene fluoride). The membranes were cultured with 5% BSA for 1 h. Primary antibodies targeting CCNB1 (Abcam, ab32053, 1:500), Bax (1:1,000, ab32503, Abcam), Bcl-2 (1:1,000, ab182858, Abcam), cleaved-caspase-3 (1:1,000, ab32042, Abcam), GLUT1 (1:1,000, ab115730, Abcam), GLUT3 (1:1,000, ab41525, Abcam), TNF-α (Abcam, ab183218, 1:1,000), IL-6 (Abcam, ab233706, 1:500), IL-1β (Abcam, ab283818, 1:1,000), GLUT4 (Abcam, ab33780, 1:500), INSR (Abcam, ab283689, 1:1,000), p-β-catenin (1:1,000, ab305261, Abcam), β-catenin (1:1,000, ab32572, Abcam), p-GSK3β (1:1,000, ab75814, Abcam), GSK3β (1:1,000, ab32391, Abcam), WNT3a (1:1,000, ab219412, Abcam), Cyclin D1 (1:1,000, ab16663, Abcam), c-Myc (1:1,000, ab32072, Abcam), and GAPDH (Abcam, ab8245, 1:3,000) were used, and then secondary antibodies were added to the membrane for 1 h and imaged after chemiluminescence treatment. The intensities of protein bands on immunoblots were measured with the ImageJ software, and the expression levels were then standardized against GAPDH as an internal reference for normalization.

### 2.9 Statistics

All data were analyzed using GraphPad Prism software (version X; GraphPad Software, San Diego, CA, USA). Results are presented as mean value ± standard deviation (SD) from at least three independent experiments. Statistical significance between groups was determined using one-way ANOVA followed by Tukey’s post-hoc test for multiple comparisons. For comparisons between two groups, an unpaired Student’s *t*-test was used. A *p*-value of less than 0.05 was considered statistically significant.

## 3 Results

### 3.1 CCNB1 is highly expressed in HG -stimulated HTR8/SVneo cells

**Figure 1:**
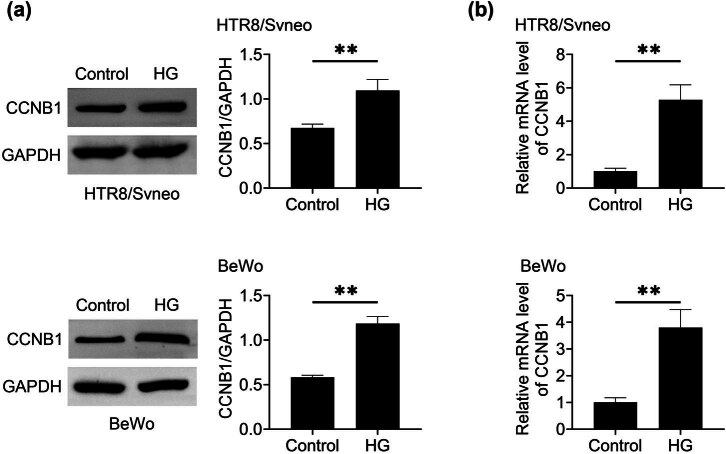
In HG -stimulated HTR8/SVneo cells, CCNB1 is highly expressed. (a) Immunoblot showed the expression of CCNB1 in control and HG stimulated HTR8/SVneo cells and BeWo cells. (b) qPCR assays showed the mRNA levels of CCNB1 in control and HG stimulated HTR8/SVneo cells and BeWo cells. **p < 0.01, HG vs control. HG, high glucose.

To evaluate the effects of CCNB1 on the progression of GDM, we first constructed a GDM cell model using trophoblasts cells, HTR8/SVneo cells and BeWo cells, upon the treatment of HG for 24 h. Afterwards, we conducted Immunoblot assays to measure the levels of CCNB1 expression. It was observed that the expression of CCNB1 was increased in HTR8/SVneo cells and BeWo cells stimulated with HG, as compared to the control group (Figure 1a). Similarly, qPCR assays indicated that CCNB1 mRNA levels were upregulated in HG-stimulated HTR8/SVneo cells and BeWo cells (Figure 1b). Therefore, CCNB1 was highly expressed in trophoblast cells of GDM model.

### 3.2 Knockdown of CCNB1 promotes the growth of HG-stimulated HTR8/SVneo cells

**Figure 2:**
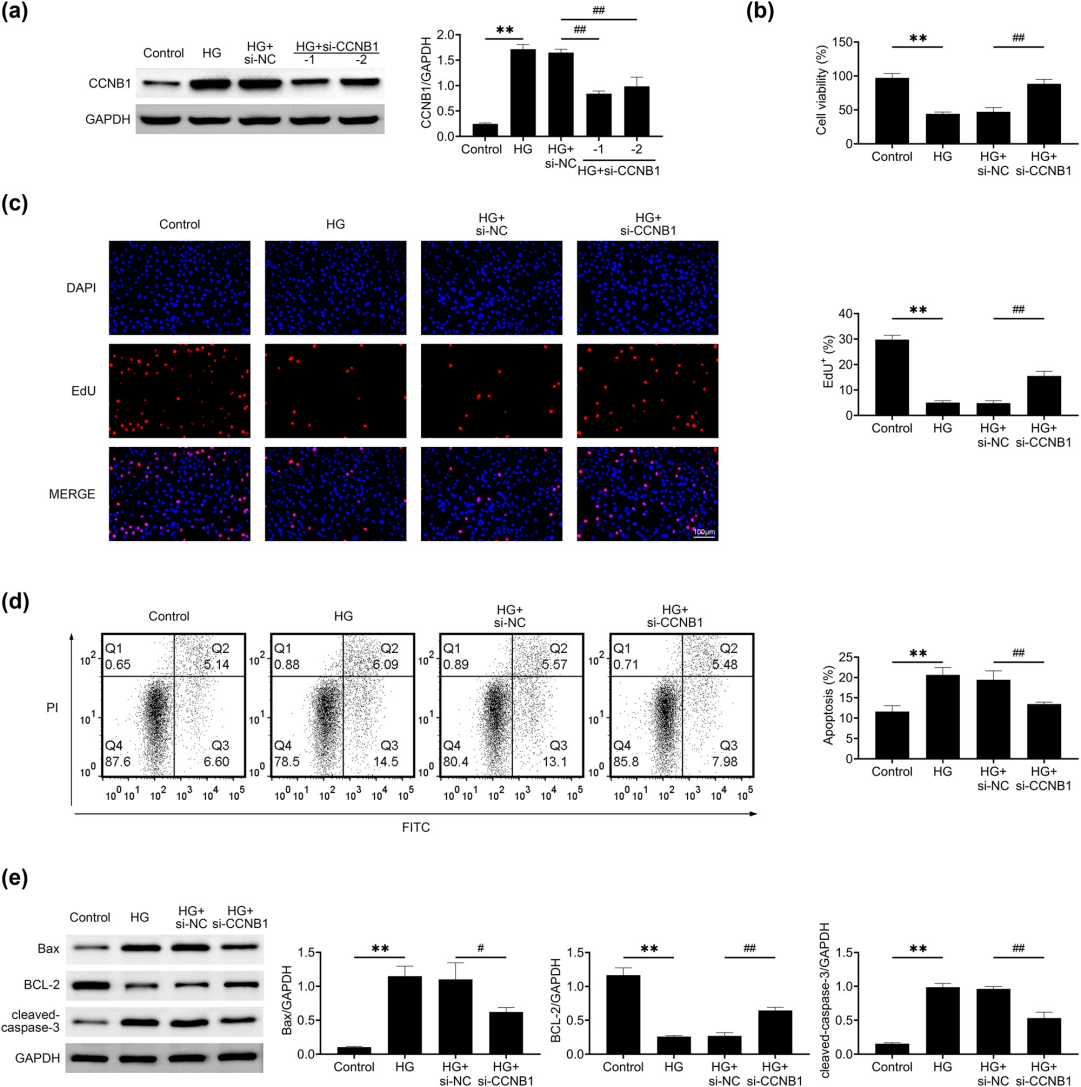
Knockdown of CCNB1 promotes the growth of HG -stimulated HTR8/SVneo cells. (a) Immunoblot showed the expression of CCNB1 in control and HG stimulated HTR8/SVneo cells upon the transfection of NC and CCNB1 siRNAs (#1 and #2). (b) CCK-8 assays showed the growth of control and HG stimulated HTR8/SVneo cells upon the transfection of NC and CCNB1 siRNAs for 24 h. The OD450 value was measured. (c) Edu assays showed the growth of control and HG stimulated HTR8/SVneo cells upon the transfection of NC and CCNB1 siRNAs for 24 h. Red panel indicates Edu-positive cells. Scale bar, 100 μm. The percentage of Edu-positive cells was measured. (d) Flow cytometry (FCM) assays showed the apoptosis of control and HG stimulated HTR8/SVneo cells upon the transfection of NC and CCNB1 siRNAs for 24 h. The percentage of apoptosis cells was measured. (e) Immunoblot assays showed the expression of Bax, Bcl-2, and cleaved caspase-3 of control and HG stimulated HTR8/SVneo cells upon the transfection of NC and CCNB1 siRNAs for 24 h. **p < 0.01, si-CCNB1 vs si-NC. HG, high glucose, NC, negative control.

Following this, siRNAs targeting CCNB1 were introduced into HTR8/SVneo cells stimulated by HG to suppress the expression of CCNB1. Immunoblot assays revealed that the transfection of both CCNB1 siRNAs significantly reduced the expression of CCNB1 in HTR8/SVneo cells under HG conditions (Figure 2a). We then used CCNB1 siRNA #1 and performed CCK-8 assays. Interestingly, the growth rates of HG-stimulated HTR8/SVneo cells were significantly increased after CCNB1 ablation, with the increased OD450 value (Figure 2b). In a similar fashion, educational assays revealed that reducing CCNB1 levels resulted in an elevated proportion of Edu-positive HTR8/SVneo cells, implying the enhancement of cellular proliferation (Figure 2c). Furthermore, we performed the FCM assays. Our data revealed that CCNB1 depletion blocked the apoptosis of HG-stimulated HTR8/SVneo cells (Figure 2d). Furthermore, the levels of apoptosis markers such as Bax and cleaved caspase-3 were found to be reduced, while Bcl-2 expression was elevated in HTR8/SVneo cells stimulated with HG, providing additional evidence of the inhibition of apoptosis (Figure 2e). Collectively, CCNB1 ablation promotes the growth of HTR8/SVneo cells.

### 3.3 Knockdown of CCNB1 suppresses inflammation in HG -stimulated HTR8/SVneo cells

**Figure 3:**
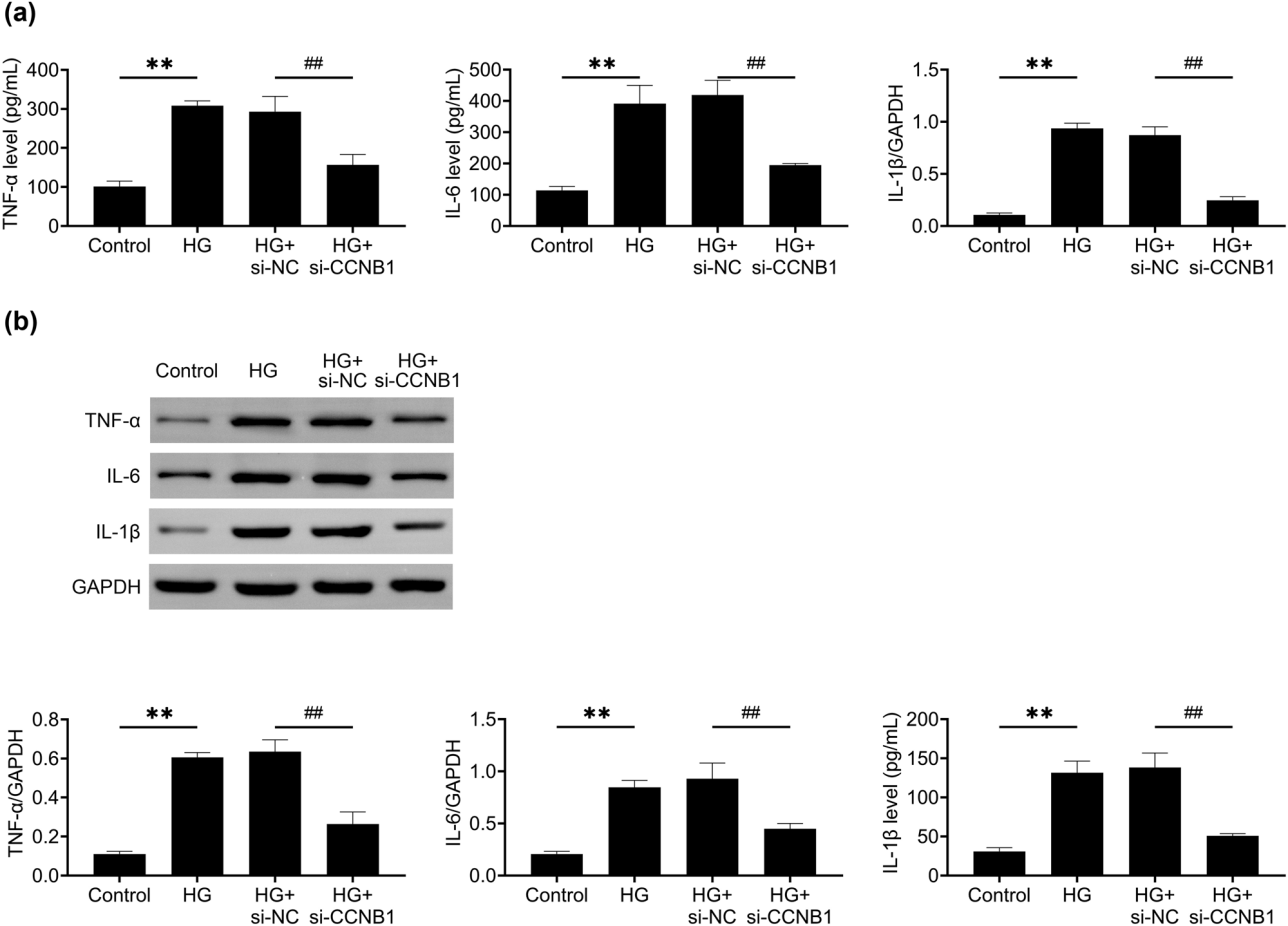
Knockdown of CCNB1 suppresses inflammation in HG -stimulated HTR8/SVneo cells. (a) ELISA showed the secretion of TNF-α, IL-6, and IL-1β of control and HG stimulated HTR8/SVneo cells upon the transfection of NC and CCNB1 siRNAs for 24 h. (b) Immunoblot assays showed the expression of TNF-α, IL-6, and IL-1β of control and HG stimulated HTR8/SVneo cells upon the transfection of NC and CCNB1 siRNAs for 24 h. **p < 0.01, si-CCNB1 vs si-NC. HG, high glucose, NC, negative control.

Next we examined how CCNB1 influences inflammation in HTR8/SVneo cells. Through ELISA assays, it was found that the absence of CCNB1 led to a reduction in the secretion of TNF-α, IL-6, and IL-1β, three key inflammatory factors, in HG-stimulated HTR8/SVneo cells (Figure 3a). Consistently, immunoblot assays also revealed that CCNB1 ablation suppressed the expression of these factors (Figure 3b). Therefore, CCNB1 depletion also restrained the inflammatory response of HTR8/SVneo cells.

### 3.4 Reducing CCNB1 expression improves glucose uptake in HG -stimulated HTR8/SVneo cells

**Figure 4:**
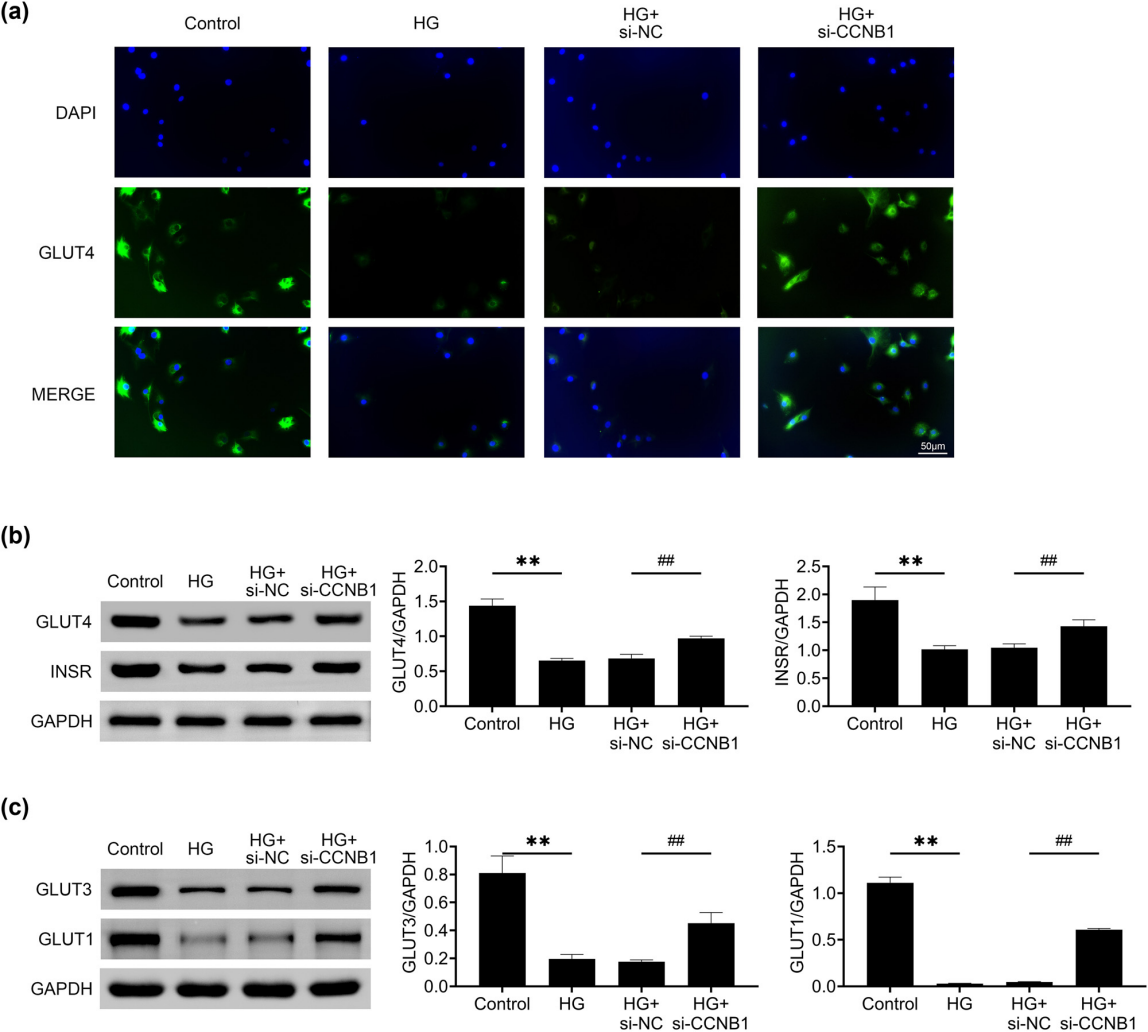
Reducing CCNB1 expression improves glucose uptake in HG -stimulated HTR8/SVneo cells. (a) Immunostaining showed the intensity of GLUT4 in control and HG stimulated HTR8/SVneo cells upon the transfection of NC and CCNB1 siRNAs for 24 h. Green panel indicates Edu-positive cells. Scale bar, 50 μm. (b) Immunoblot assays showed the expression of GLUT4 and INSR of control and HG-stimulated HTR8/SVneo cells upon the transfection of NC and CCNB1 siRNAs for 24 h. (c) Immunoblot assays showed the expression of GLUT3 and GLUT1 of control and HG stimulated HTR8/SVneo cells upon the transfection of NC and CCNB1 siRNAs for 24 h. **p < 0.01, si-CCNB1 vs si-NC. HG, high glucose, NC, negative control.

We then clarified the effects of CCNB1 on the glucose uptake of HTR8/SVneo cells upon HG incubation. Immunoblot analysis revealed that knockdown of CCNB1 using its siRNAs led to elevated GLUT4 expression in HG-stimulated HTR8/SVneo cells, as evidenced by the enhanced signal intensity of GLUT4 bands (Figure 4a). Additionally, Immunoblot assays revealed an upregulation in the expression of GLUT4 and INSR in HTR8/SVneo cells treated with HG, indicating enhanced glucose uptake (Figure 4b). In addition, the expression of GLUT3 and GLUT1 was increased in HG-stimulated HTR8/SVneo cells, further confirming the conclusion (Figure 4c). Therefore, CCNB1 knockdown improved glucose uptake of HTR8/SVneo cells.

### 3.5 CCNB1 depletion inhibits the Wnt/β-catenin pathway in HG-stimulated HTR8/SVneo cells

**Figure 5:**
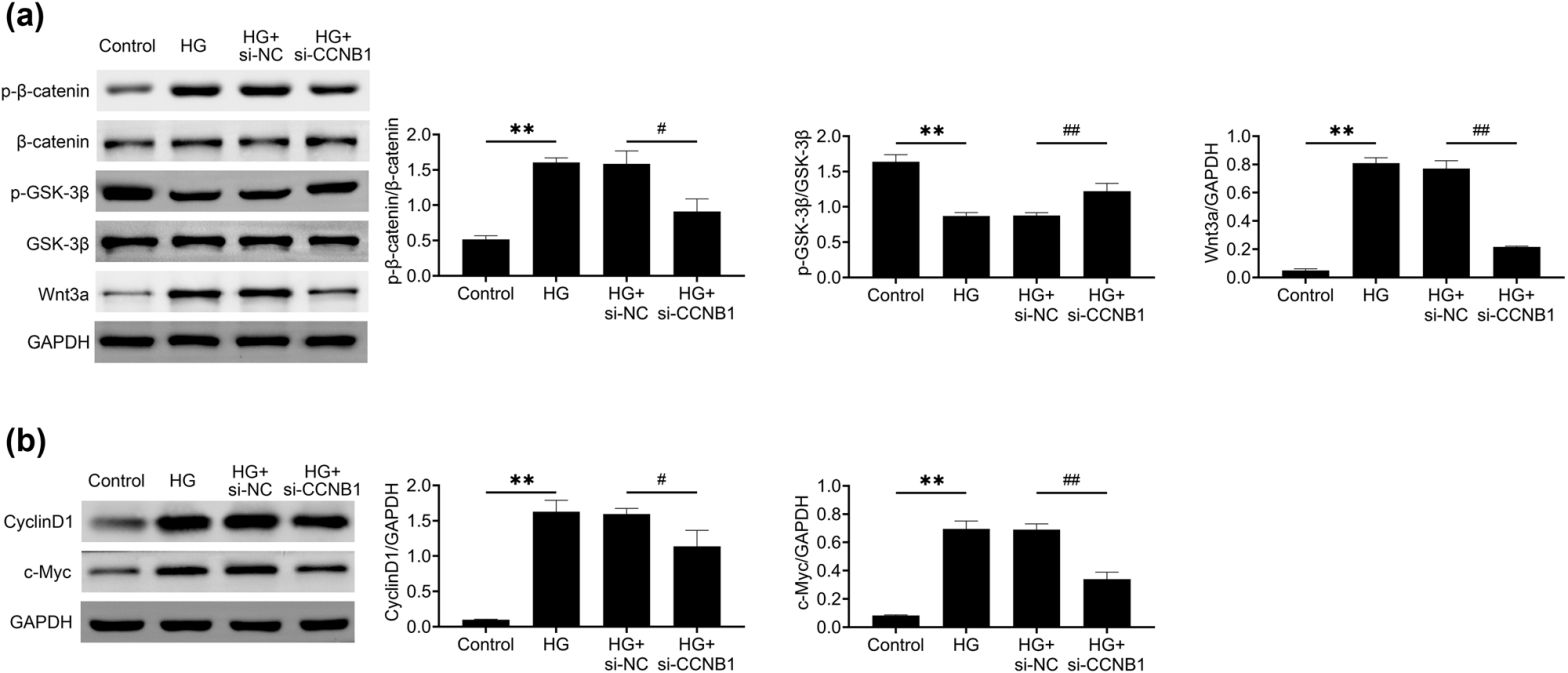
CCNB1 depletion inhibits the Wnt/β-catenin pathway in HG -stimulated HTR8/SVneo cells. (a) Immunoblot assays showed the expression and phosphorylation levels of β-catenin and GSK3β and expression of Wnt3α of control and HG-stimulated HTR8/SVneo cells upon the transfection of NC and CCNB1 siRNAs for 24 h. (b) Immunoblot assays showed the expression levels of Cyclin D1 and C-Myc of control and HG-stimulated HTR8/SVneo cells upon the transfection of NC and CCNB1 siRNAs for 24 h. **p < 0.01, si-CCNB1 vs si-NC. HG, high glucose, NC, negative control.

Next we elucidated how the reduction in CCNB1 inhibits the progression of GDM. A previous study highlighted the significance of the Wnt/β-catenin pathway in GDM [7]. Then, we detected the effects of CCNB1 in HG-stimulated HTR8/SVneo cells. Immunoblot showed the expression and phosphorylation levels of β-catenin and GSK3β, and expression levels of Wnt3a, 3 regulators of this pathway, in HTR8/SVneo cells. Surprisingly, the levels of β-catenin phosphorylation were found to decrease, while the phosphorylation levels of GSK3β increased in HTR8/SVneo cells stimulated by HG. Additionally, there was a downregulation in the expression of Wnt3a, indicating a possible inhibition of the Wnt/β-catenin pathway (Figure 5a). Further, the expression levels of downregulators, Cyclin D1, and c-Myc, were decreased in HG-stimulated HTR8/SVneo cells (Figure 5b). Therefore, knockdown of CCNB1 suppressed Wnt/β-catenin NF-κB pathway in HG-induced HTR8/SVneo cells.

## 4 Discussion

GDM is a prevalent issue during pregnancy, and it is widely recognized that dysfunctional trophoblasts play a role in unfavorable pregnancy results [6]. The presence of elevated glucose levels in the uterus hinders the proper development, infiltration, growth, and programmed cell death of trophoblasts, leading to a decline in placental effectiveness [18]. In this study, we explored the role of CCNB1 in HG-induced trophoblast dysfunction and examined whether its knockdown could alleviate adverse cellular responses through the Wnt/β-catenin signaling pathway.

Our findings revealed that CCNB1 is significantly upregulated in HG-stimulated HTR8/SVneo cells compared to controls, consistent with previous reports of elevated CCNB1 expression in diabetic mice. The increased expression of CCNB1 under HG conditions implies the significant involvement of this protein in the abnormal reaction of trophoblasts to hyperglycemia. Furthermore, heightened levels of CCNB1 have been detected in different types of cancers like HCC, breast cancer, and bladder cancer, suggesting that elevated glucose levels could induce comparable growth signals in trophoblast cells [9,14]. Specifically, CCNB1 is known to facilitate cell cycle control and DNA synthesis in HCC, enhancing the proliferation of cancer cells [9]. Similarly, in trophoblasts, abnormal CCNB1 expression is associated with spontaneous abortion, as circ-CCNB1 regulates trophoblast proliferation and invasion via the miR-223/SIAH1 axis.

Knocking down CCNB1 led to a significant contribution in cell viability and proliferation while suppressing apoptosis in HG-stimulated HTR8/SVneo cells. This underscores the pivotal role of CCNB1 in trophoblast growth and survival under hyperglycemic conditions. The significance of the pro-apoptotic impact of reducing CCNB1 lies in its relevance to placental dysfunction in GDM due to its association with inhibited trophoblast apoptosis. In GDM, trophoblast dysfunction induced by mucin1 is known to result in heightened apoptosis through the Wnt/β-catenin pathway, illustrating the importance of promoting apoptosis [7]. Therefore, by targeting CCNB1, it is possible to potentially enhance trophoblast functionality and enhance placental well-being in GDM.

Inflammation is a hallmark of GDM-associated trophoblast dysfunction [19]. Our study found that CCNB1 knockdown significantly reduced the levels of pro-inflammatory cytokines, such as TNF-α, IL-6, and IL-1β, in HG-stimulated HTR8/SVneo cells. This suggests that CCNB1 plays a role in mediating the inflammatory response in trophoblast cells, possibly via interactions with key signaling pathways like NF-κB or STAT3. Reducing inflammation in the placenta could improve pregnancy outcomes by decreasing the risk of preeclampsia, stillbirth, and other complications associated with GDM.

Exposure to elevated levels of glucose hinders the uptake of glucose in trophoblast cells, worsening the negative metabolic consequences of GDM [20]. Our data demonstrate that CCNB1 knockdown improves glucose uptake in HG-stimulated HTR8/SVneo cells by enhancing GLUT4 expression and INSR protein levels. This suggests that targeting CCNB1 could restore glucose uptake and metabolism in trophoblasts, ultimately improving placental function and fetal development.

The Wnt/β-catenin pathway plays a critical role in the normal formation of the placenta and is also involved in the development of GDM [7]. Our results reveal that CCNB1 knockdown inhibits the activation of the Wnt/β-catenin pathway in HG-stimulated HTR8/SVneo cells, as evidenced by reduced β-catenin and p-β-catenin levels. The increased phosphorylation of GSK3β following CCNB1 knockdown may indicate a compensatory mechanism within the Wnt/β-catenin pathway. This feedback regulation could be essential for maintaining cellular homeostasis under conditions of altered cell cycle dynamics, such as those observed with CCNB1 depletion. By downregulating this pathway, CCNB1 knockdown may reduce trophoblast proliferation and inflammation while enhancing glucose uptake and differentiation, thereby mitigating the adverse effects of GDM on placental health. Dietrich et al. have shown that the Wnt/β-catenin pathway is essential for trophoblast differentiation and invasion, supporting the critical role of this pathway in placental development. Although our results suggest that CCNB1 knockdown affects both Wnt and NF-κB pathways, there is no direct evidence in this study demonstrating a mechanistic link between the two. Additional studies are required to delve into the potential interplay or communication between these signaling pathways in the presence of elevated glucose levels. Subsequent research will delve into the effects of reducing CCNB1 on additional essential metabolic controllers like AMPK, HK2, and FASN, in order to gain a more comprehensive understanding of its broader involvement in metabolic control during gestational diabetes. Moreover, further investigations will probe the possible interaction between the Wnt/β-catenin pathway and other crucial signaling pathways, such as NF-κB or STAT3, to enhance our overall comprehension of the molecular mechanisms at play when CCNB1 is reduced in trophoblast malfunction.

Given the significant role of CCNB1 in mediating trophoblast dysfunction in GDM, targeting this protein could offer a novel therapeutic strategy for managing GDM-associated placental complications. By suppressing CCNB1, there is a potential to revive trophoblast function, diminish inflammation, and enhance glucose absorption, thereby mitigating the chances of negative pregnancy consequences. Nonetheless, additional clinical investigations are necessary to confirm these results and evaluate the safety and effectiveness of CCNB1-focused treatments in patients with GDM. Furthermore, studies should explore whether targeting other cell cycle regulators, such as CDK1, in combination with CCNB1 could offer synergistic therapeutic effects.

Despite providing valuable insights into the role of CCNB1 in GDM-associated trophoblast dysfunction, this study has certain limitations. First, the use of a single trophoblast cell line (HTR8/SVneo) may not fully represent the diversity of trophoblast responses *in vivo*. Second, the *in vitro* nature of the study limits the ability to assess the systemic effects of CCNB1 knockdown on pregnancy outcomes. Future research should include *in vivo* models and clinical samples to corroborate these findings. Additionally, the molecular mechanisms underlying the interactions between CCNB1 and other signaling pathways, such as NF-κB, require further investigation.

Our results differ from the research conducted by Yuan et al., who found that reducing CCNB1 expression hinders the growth of cancer cells. This discrepancy could be attributed to variations in cell types and experimental conditions. Specifically, the suppression of CCNB1 in trophoblast cells exposed to high levels of glucose might trigger alternative pathways, such as the Wnt/β-catenin pathway, which could stimulate cell proliferation.

In summary, this investigation illustrates that suppressing CCNB1 can alleviate dysfunction in trophoblast cells induced by HG by regulating the Wnt/β-catenin signaling pathway. These outcomes underscore the potential therapeutic benefits of targeting CCNB1 to address complications associated with GDM and enhance pregnancy outcomes.
